# International Collaboration on Palliative Care Development Between ASCO and the Land of Hornbills

**DOI:** 10.1200/GO.22.00351

**Published:** 2023-01-11

**Authors:** Yin Yee Wong, Aaron Zi Jian Chiew, Vanessa Eaton, Frank D. Ferris, Megan Kremzier, Boon Leong Lim, Winnie Hui Yee Ling, Asri Said, Vanessa Sarchet, Tung Hui Tiong, Pei Jye Voon, Yoke Ling Choo

**Affiliations:** ^1^ Radiotherapy, Oncology and Palliative Care Department, Sarawak General Hospital, Sarawak, Malaysia; ^2^ American Society of Clinical Oncology, Alexandria, VA; ^3^ OhioHealth, Columbus, OH; ^4^ Selayang Hospital, Batu Caves, Selangor, Malaysia; ^5^ Two Tree Lodge Hospice Kuching, Sarawak, Malaysia; ^6^ Normah Medical Specialist Center, Kuching, Sarawak, Malaysia; ^7^ Faculty of Medicine and Health Sciences, Universiti Malaysia Sarawak, Sarawak, Malaysia; ^8^ Sarawak Heart Center, Kuching, Malaysia; ^9^ National Cancer Society of Malaysia Sarawak Branch, Kuching, Malaysia; ^10^ Timberland Medical Center, Kuching, Sarawak

## Abstract

Palliative care in Sarawak is mainly provided by health care professionals with limited formal training in palliative care. Therefore, in 2020, collaborative work between Sarawak General Hospital, University Malaysia Sarawak, and ASCO began. This study reports on the outcome of this collaboration.

**METHODS:**

The collaboration was initiated with the first ASCO Palliative Care e-course, Train the Trainer program, International Development and Education Award—Palliative Care and translation of ASCO Palliative Care Interdisciplinary Curriculum resources.

**RESULTS:**

This collaboration has resulted in the change of practice of palliative care among the oncology team of Sarawak General Hospital.

**CONCLUSION:**

It encourages more timely palliative care referrals to ensure that patients with complex physical, psychosocial, and spiritual needs have the necessary input and support from the palliative care team throughout the course of patients’ illnesses.

## INTRODUCTION

Sarawak, nicknamed the Land of Hornbills, located on northwest Borneo Island is the largest and fourth most populated state of Malaysia. It spans across 120,000 square kilometers hosting 2.9 million population.^1^ As of 2010, 54% of the population is urban while 46% are residing in rural areas.^2^ Given its widespread geographical area, Sarawak has the most dispersed rural communities in Malaysia.^2^ Despite the progressive development of infrastructure in Sarawak, inadequate connectivity in the rural area with consequential accessibility issues especially for health care service remains a major challenge in the state.

**CONTEXT**

**Key Objective**
This report highlights the impact of the international collaborative work between ASCO and Sarawak.
**Knowledge Generated**
The collaborative effort meets the palliative care educational needs which resulted in positive changes in practice of the oncology department of Sarawak General Hospital. Trainer skills were taught in the trainer program, building a reservoir of local health care champions to ensure sustainability of palliative care service in Sarawak. The collaborative effort also led to translational work of the education materials, ensuring language is not a barrier in learning palliative care.
**Relevance**
All these efforts are integral to the development in Sarawak to be a center of excellence for palliative care.

Challenges in providing palliative care in Sarawak are many and include lack of trained personnel, lack of awareness and buy-ins on palliative care service, financial constraints, geographical challenge, and limited access to health care service in rural areas. To our knowledge, to date, there is only one palliative care physician in the state of Sarawak since mid-2021. Therefore, palliative care services are often provided by doctors and nurses who are passionate in palliative care, many with limited formal training in palliative care.

Sarawak General Hospital (SGH) is a tertiary public hospital and the only oncology center in the state which also serves as a teaching hospital for University Malaysia Sarawak (UNIMAS). Palliative care service in SGH has been uniquely initiated and developed under the leadership of the oncology unit. The services provided include a nine-bedded inpatient ward, intrahospital and interhospital consult services, outpatient palliative care clinic, and combined multidisciplinary motor neuron disease outpatient clinic.

In addition, there are several independent, nonprofit organizations, namely National Cancer Society (Sarawak Branch), Two Tree Lodge Hospice and Kuching Life Care Society providing support and palliative care services in the community. The close-knitted collaboration between hospital-hospices enables patients who live within a 20-km radius to have seamless transition into the community, back to the comfort of their home and vice versa when needed.

ASCO International Cancer Corps (ICC) was first introduced to the oncology SGH team by Malaysian Oncological Society in 2019. ICC is designed to improve the quality of cancer care of medical institutions in low- and middle-income countries, addressing three primary areas of need namely, multidisciplinary management of common cancers, integration of palliative care into cancer care, and improvement of quality of care using evidence-based quality measures.

In 2020, collaborative work between SGH, UNIMAS, and ASCO began with the signing of Memorandum of Understanding. Unforeseen emergence of COVID-19 pandemic has resulted in setbacks such as scheduling, in-person training, and onsite visits of the collaboration. Nonetheless, this did not deter the team from successfully initiating the first online palliative care educational curriculum in 2021.

## METHODS

### Objectives of Palliative Care Collaboration

1. To advocate and promote palliative care awareness, competency and service development in Sarawak
2. To provide holistic palliative care through unified efforts by all health care providers and independent, nonprofit organizations, with optimal usage of available resources to provide a support system for patients and family members.
3. To achieve optimal pain and symptom control in patients with advanced cancer and ensure safe and appropriate opioid administration and titration.
4. To educate and conduct workshops on cancer pain and symptom management to empower health care providers with palliative care knowledge and skill sets via efficient and cost-effective training programs.
5. To develop trainer skills among local health care champions to expand training and educational programs and ensure sustainable service provision.

### Report of Palliative Care Collaborations

#### ASCO Palliative Care e-Course 2021.

**FIG 1:**
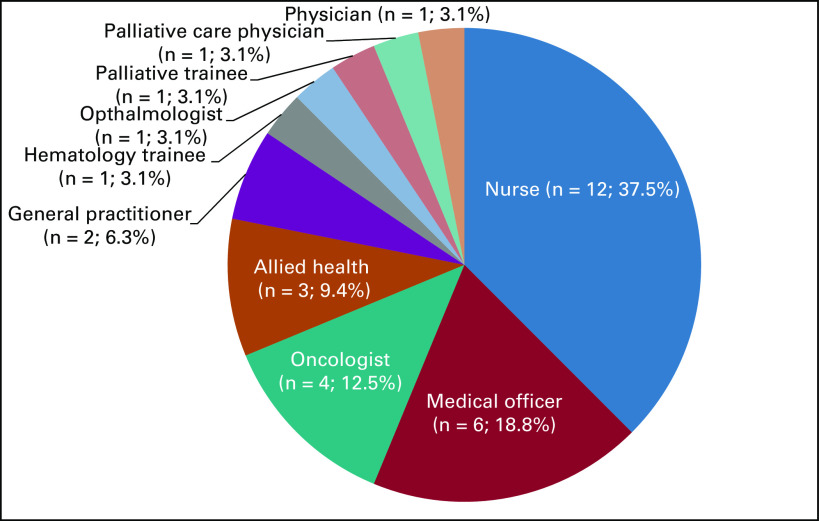
Composition of participants by profession.

The collaborative initiative between Sarawak, UNIMAS, and ASCO kicked off with the rollout of the first ASCO Palliative Care e-Course (APCeC) titled “A Taste of Palliative Medicine” in March to June 2021 attended by 32 participants (Fig 1) across Sarawak, with an average of 6.2 years' experience in palliative care. This was a 12-session comprehensive and personalized online course conducted weekly with participants having access to precourse self-study material, namely PowerPoints notes, Palliative Care Interdisciplinary Curriculum (PCIC) videos, and related journals/articles. The course featured case-based presentations and breakout sessions on different aspects of palliative care.

The main objectives of the course are

1. Understanding the concepts and principles of palliative and end-of-life care.
2. Effective communication with patients and their families.
3. Optimal pain and symptom control including safe and effective opioids usage and titration
4. Networking of palliative care providers across Sarawak

**FIG 2:**
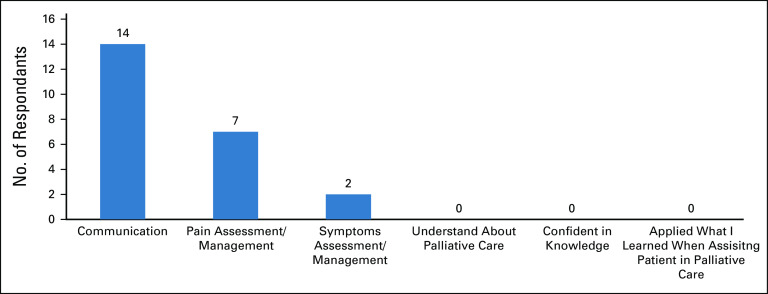
Practice change in daily work after course.

A postcourse impact assessment was done 6 months later, with 22 of 32 participants answering the survey voluntarily, representing 69% of attendees.^3^ All respondents (n = 22) reported that they were able to provide and use the skills learned in their daily practice. The most reported changes were related to communication, pain, and symptoms assessment/management (Fig 2). Seventy-one percent of respondents stated that they made these changes within 4 weeks of attending the course. Overall, of those who responded to the follow-up impact assessment, the results suggested that the course was successful.

#### Train the Trainer Program 2022.

**FIG 3:**
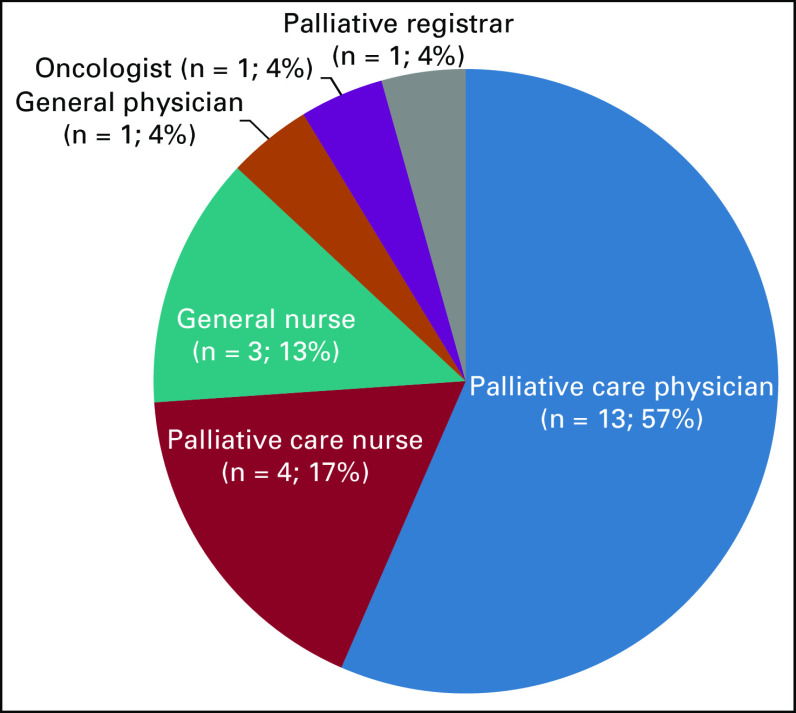
Composition of participants by profession.

After the successful first APCeC course, a weekly virtual Train the Trainer (TTT) course was conducted over a span of 3 weeks from February 2022 to March 2022. There were a total of 27 participants, primarily palliative care physicians, and palliative care nurses across Sarawak and other parts of Malaysia (Fig 3). The participants have an average of 6.9 years of experience in their current profession, with respondents reported giving training to up to 100 participants annually and conducting up to 12 trainings per year.

The objectives of the course were to equip participants to effectively

1. Present information.
2. Facilitate discussions.
3. Provide feedback.

A postcourse evaluation survey obtained from 23 respondents, representing 85% of all participants showed that 87% (n = 20) reported an increase in their ability to present information effectively, 91% (n = 21) reported an increase in ability to facilitate discussion effectively, and 83% (n = 19) reported an increase in ability in providing feedback effectively.^4^ These results suggested that the course has further enhanced the ability of respondents to be trainers and to present information, provide feedback, and facilitate discussion effectively.

#### International Development and Education Award—Palliative Care.

International Development and Education Award—Palliative Care award from the Conquer Cancer Foundation encompasses 3 years of complimentary membership to ASCO, attendance to ASCO's 2022 Annual Meeting in Chicago, TTT Workshop, mentorship program, and extended tour award to University of Chicago. It also provides a platform for networking and future collaborations among award recipients. Dr Wong Yin Yee, palliative care unit registrar of SGH and one of the local palliative care champions is the first recipient from Malaysia of this globally competitive award.

In July 2022, Dr Wong Yin Yee, along with other awardees of International Development and Education Award—Palliative Care, were able to attend an onsite TTT workshop by Dr Frank Ferris and team in Chicago. Essential trainer skills including providing effective feedback and elevator pitches were imparted, and there was plenty of opportunity to practice these skills during the course.

Furthermore, the mentorship program creates opportunities for mentor and mentee to work on projects to improve palliative care outcomes. The cultural exchange provided a platform for sharing of ideas, bench markings, support, and opportunity for future collaboration.

#### Translation of ASCO PCIC Resources.

ASCO PCIC has an extensive and comprehensive educational curriculum available in 10 languages to date. During the first APCeC virtual course, some participants reported that they had trouble following the precourse videos because of language limitations as the primary language in Malaysia is Malay, while English is the secondary language. Therefore, major efforts are underway to translate these resources to Malay language, to ensure that language is not a barrier in learning palliative care. The translations are done in partnership with developers of the curricular materials. They were guided by Malaysian palliative care providers from various states who volunteered their time and expertise to ensure translational accuracy and availability of resources.

## RESULTS

Because of limitations in resources and manpower, the standard practice for referrals to the palliative care team in SGH is largely for patients on best supportive care. APCeC helps to provide a fundamental framework for palliative care education that is invaluable in equipping oncologists and oncology trainees with the necessary knowledge and skill sets to better identify and meet palliative care needs among their patients. It ensures a more competent and timely palliative care provision at a general level by the oncology team of SGH and enables the team to incorporate basic palliative care management early in the course of illness alongside active oncological treatment.

**FIG 4:**
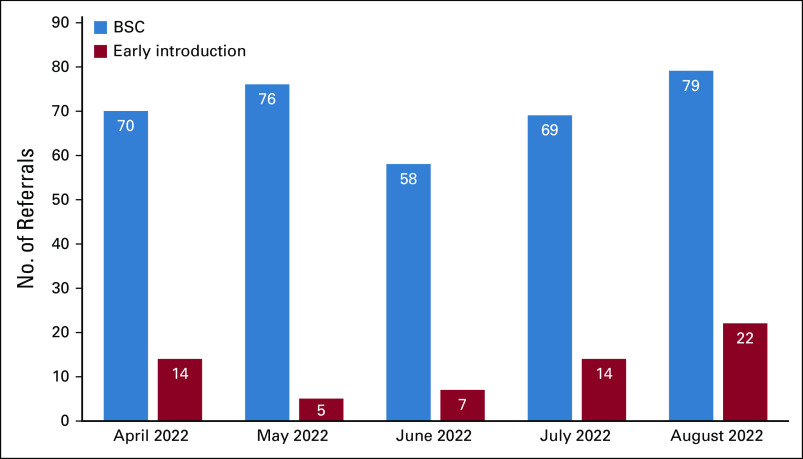
Reasons for referral. BSC, best supportive care.

With the availability of the first palliative care physician at SGH since mid-2021, the palliative care team has opened for earlier referrals for oncology patients still undergoing active oncological treatment with more complex palliative care needs as observed in Figure 4. These early referrals are known as early introduction. The ASCO collaboration enhances teamwork and helps the oncology team to recognize their limitations while providing general palliative care, thereby encouraging more timely palliative care referrals when appropriate to ensure that patients with more complex physical, psychosocial, and spiritual needs have the necessary input and support from the palliative care team throughout the course of patients' illnesses.

## DISCUSSION

The palliative care international collaboration with ASCO ICC was set in motion with virtual meetings to understand local challenges and the palliative care educational needs of Sarawakians, followed by elaborate discussions on training objectives and intended outcomes. These had led to the development of a highly personalized palliative care education program which is one of the highlights of this collaboration.

The first APCeC program allowed participants from various districts across Sarawak to gain knowledge and skills effectively in the comfort of their home. This eliminated financial costs for travel, accommodation, and time away from work. The 12-session e-course spanned out across a few months, allowing participants to read up materials and learn and digest information at a reasonable pace. The successful rollout of the first APCeC program had sparked interest among palliative care providers from the Malaysia Ministry of Health. Subsequently, it has led to meaningful developments such as the TTT program and second APCeC program.

The TTT program was developed to address the need to equip and nurture trainer skills among local palliative care providers so as to ensure high quality of palliative care education and sustainability in training. The program benefitted not only Sarawak palliative care providers but also included palliative care providers across Malaysia. The TTT program encouraged participants to effectively practice the skills taught in small breakout groups, namely presentation, facilitating, and feedback skills.

Meanwhile, the second APCeC program is a collaborative effort not only between ASCO, UNIMAS, and SGH but also involves Malaysia Ministry of Health. Facilitators from the ASCO team were paired with Malaysian cofacilitators, namely palliative care providers who had completed the TTT program. There was an overwhelming response with 100 applications for a limited 60 slots. The second APCeC was similarly a 12-session comprehensive virtual course that concluded in August 2022. There were 30 participants from Sarawak and 30 participants from other states in Malaysia.

Some of the challenges experienced working on international collaboration includes scheduling, cultural differences, language, and technology failure. Scheduling for discussion, preparation, and delivery of course calls for dedication and commitment beyond working hours by both international and local teams. Although cultural differences are present, robust discussion in smaller breakout groups and conscientiously obtaining feedback at the end of every session provided opportunity to discuss on adaptation of skills learnt to local setting.

The official and national language of Malaysia is Malay while English is the second language. Communication sessions during break out groups may remain a challenge for participants whose first or second language is not English. Technology failures experienced by some participants are mainly band width issues causing inability to turn on videos throughout the entire course or video lag. Participants are still able to listen to lectures and participate through chat boxes and voice communication.

Despite the challenges, the international ASCO ICC collaboration, with aligned goals and objectives, has brought about tremendous positive impacts. Efforts made throughout the collaboration by both parties are not only worthwhile but also enriching.

The reported outcomes for the first APCeC program suggest that it was a success. It is worth noting that the majority of participants have basic background palliative care knowledge and are actively involved with palliative care work. Hence, they may be more receptive to the contents delivered and more able to put knowledge and skills learnt into practice in a supportive work environment. Therefore, further evaluation on the outcome of the second APCeC program is crucial as the participants were from a more diverse nonpalliative background with close to one third of the participants reported not having any prior exposure to palliative care. The evaluation would provide a better insight on the impact and value of the e-courses. This will enable the team to evaluate and explore different learning needs and adapt the e-course contents to cater to different groups of participants accordingly.

Moving forward, the SGH palliative care team is anticipating the in-person training by Dr Frank Ferris and his team of experts from ASCO in November 2022 to further enhance the skill set taught during the APCeC program. The esteemed ASCO palliative care experts will also conduct an in-person second TTT workshop focusing on advocacy, leadership and mentoring during their anticipated visit.

In conclusion, the reproducibility of the comprehensively crafted APCeC curriculum along with the abundant resources from ASCO will serve as an invaluable and pivotal platform for the expansion of palliative care service in various departments of SGH and across districts in Sarawak. In addition, the TTT program will equip and enable local leaders in the fraternity to contribute to palliative care education and development in Sarawak and Malaysia.

These international collaborations are integral to the development of palliative care in Sarawak, with the vision to build Sarawak to be the center of excellence for impeccable palliative care service, education and training in Malaysia and beyond.
